# Analysis of prognosis and background liver disease in non-advanced hepatocellular carcinoma in two decades

**DOI:** 10.1371/journal.pone.0297882

**Published:** 2024-03-07

**Authors:** Shun Kaneko, Yasuhiro Asahina, Miyako Murakawa, Seishin Azuma, Kento Inada, Tomohiro Mochida, Keiya Watakabe, Taro Shimizu, Jun Tsuchiya, Masato Miyoshi, Fukiko Kawai-Kitahata, Sayuri Nitta, Marie Takahashi, Tomoyuki Fujioka, Mitsuhiro Kishino, Tatsuhiko Anzai, Sei Kakinuma, Mina Nakagawa, Ryuichi Okamoto

**Affiliations:** 1 Department of Gastroenterology and Hepatology, Tokyo Medical and Dental University, Tokyo, Japan; 2 Department of Liver Disease Control, Tokyo Medical and Dental University, Tokyo, Japan; 3 Department of Gastroenterology and Hepatology, Tokyo Metropolitan Bokutoh Hospital, Tokyo, Japan; 4 Department of Diagnostic Radiology and Nuclear Medicine, Tokyo Medical and Dental University, Tokyo, Japan; 5 Department of Biostatistics, M&D Data Science Center, Tokyo Medical and Dental University, Tokyo, Japan; Kindai University Faculty of Medicine, JAPAN

## Abstract

**Background/Aim:**

Antiviral hepatitis and systemic therapies for hepatocellular carcinoma (HCC) remarkably progressed in the recent 10 years. This study aimed to reveal the actual transition and changes in the prognosis and background liver disease in non-advanced HCC in the past 20 years.

**Methods:**

This retrospectively recruited 566 patients who were diagnosed with non-advanced HCC from February 2002 to February 2022. The prognosis was analyzed by subdividing according to the diagnosis date (period I: February 2002–April 2009 and period Ⅱ: May 2009–February 2022).

**Results:**

Patients in period II (n = 351) were significantly older, with lower albumin-bilirubin (ALBI) scores and alpha-fetoprotein (AFP) and more anti-viral therapy, systemic therapy, and hepatic arterial infusion chemotherapy as compared with those in period I (n = 215). The etiology ratio of the background liver disease revealed decreased hepatitis C virus from 70.6% to 49.0% and increased non-B, non-C from 17.7% to 39.9% from periods I to Ⅱ. The multivariate analysis revealed older age and higher ALBI score in Barcelona Clinic Liver Cancer (BCLC) 0/A stage, AFP of >20 ng/mL, and higher ALBI score in BCLC B stage as independent prognosis factors. Fine-Gray competing risk model analysis revealed that liver-related deaths significantly decreased in period II as compared to period I, especially for BCLC stage 0/A (HR: 0.656; 95%CI: 0.442–0.972, *P* = 0.036).

**Conclusion:**

The characteristics of patients with non-advanced HCC have changed over time. Appropriate background liver management led to better liver-related prognoses in BCLC 0/A.

## Introduction

Liver cancer is the fourth leading cause of cancer-related death worldwide, and the World Health Organization estimates that more than 1 million people will die from the disease by 2030. Hepatocellular carcinoma (HCC) is leading cancer, accounting for 90% of liver cancer, and remains a global threat [1, 2]. The annual report of the national cancer institute reported the increased incidence rate of liver cancer in females [3].

Antiviral hepatitis therapy remarkably progressed, especially for hepatitis B and C. The effectiveness of direct-acting antivirals (DAAs) in eradicating the hepatitis C virus (HCV) is firmly established and is one of the greatest successes of medical therapeutics in the last 20 years [4]. Additionally, antiviral treatment for hepatitis B virus (HBV) with either pegylated interferon or a nucleos(t)ide analog (lamivudine, adefovir, entecavir, tenofovir disoproxil, or tenofovir alafenamide) reduce the risk of HCC and HBV-associated mortality in patients with chronic HBV infection [5, 6]. Mortality rates have started to decrease, especially in Asia and Southern Europe, as a result of efforts to control HBV and HCV infection [7].

Furthermore, systemic therapy for advanced HCC remarkably progressed. Randomized, double-blind, placebo-controlled trials in the late 2000s revealed the survival benefit of sorafenib with advanced HCC [8]. Since the mid-2010s, multiple phase III trials of molecular target agents (MTAs) successfully demonstrated efficacy [9–12]. Five MTAs have been approved for advanced HCC, with lenvatinib showing noninferiority in terms of survival benefit as compared with sorafenib in the frontline setting, and with regorafenib, cabozantinib, and ramucirumab demonstrating superiority to placebo in the second line setting after sorafenib. The combination therapy of atezolizumab and bevacizumab was approved because of its ability to significantly prolong the progression-free survival and overall survival (OS) in patients with unresectable HCC [13]. Indications for systemic therapy tend to extend to earlier stages, as in Barcelona Clinic Liver Cancer (BCLC) stage B [14]. Disorderly repetition of transcatheter arterial chemoembolization (TACE) after the recurrence of intermediate-stage HCC is not only less effective but will also worsen liver function. Systemic therapy for patients who are refractory or unsuitable for TACE is considered as well as for patients with advanced stage HCC [14, 15]. Therefore, migration from TACE to systemic therapy at the appropriate timing has been recommended [16, 17].

Thus, the systemic therapy for HCC has dramatically progressed along with the treatment of background liver diseases. Some cases can be expected with long-term survival following these progresses, but cases with poor prognoses remain. Optimizing the treatment of HCC by evaluating the tumor burden and background liver disease is important while a variety of treatment methods and processes exists. Though there was time-dependent study on systemic therapy for advanced HCC [18], studies on non-advanced HCC were not well conducted considering the progress of background liver disease therapy and systemic therapy. Therefore, we focus on non-advanced HCC.

This study aimed to reveal the actual transition and changes in the prognosis and background liver disease of non-advanced HCC in the past 2 decades. Additionally, we review and reveal the optimal treatment strategy for HCC.

## Methods

### 1. Patients

Patients with HCC were retrospectively enrolled from 705 patients who were diagnosed at the Tokyo Medical and Dental University (TMDU) Hospital from February 2002 to February 2022. HCC diagnosis was based on pathologically proven HCC or radiologic findings, such as typical tumor arterial enhancement followed by a washout pattern in the portal venous phase or the equilibrium phase on dynamic computed tomography (CT) or magnetic resonance imaging (MRI), following the criteria of practice guidelines [16, 17, 19]. Angiography which included CT during hepatic arteriography and arterial portography was performed mainly prior to 2008 (EOB-MRI was not available) to obtain a definitive HCC diagnosis or in the course of TACE treatment.The non-advanced stage HCC was defined with the BCLC staging system [14]. We defined BCLC 0/A/B-stage as non-advanced stage HCC without either extrahepatic metastasis, macrovascular invasion, or ECOG performance status ≥1. Tumor burden was calculated from tumor number and maximum diameter. Of these, 139 patients were excluded due to short follow-up periods (<1 month) and/or missing data about blood tests and treatment. Sixteen patients with advanced stages were excluded. Finally, 566 patients with non-advanced HCC were included. The Ethical Committee of TMDU Hospital approved this study, which was conducted following the 2013 revision of the Declaration of Helsinki (confirmation number: M2022-061).

Serum albumin was measured with BCP assay. Child-Pugh (C-P) classification, albumin-bilirubin (ALBI), and modified ALBI (mALBI) grades were used for liver function assessment. ALBI score was calculated based on serum albumin and total bilirubin values as previously reported [20]. ALBI grade was defined as grade 1, ≤–2.60; grade 2, >–2.60 to ≤–1.39; and grade 3, >–1.39. Furthermore, ALBI grade 2 was divided into two subgrades (2a and 2b) using a cut-off value (ALBI score of –2.270), and such ALBI grades were defined as mALBI grade [21].

We subdivided the patients with non-advanced HCC into two groups according to the date of diagnosis and hospitalization (period I: February 2002–April 2009 and period Ⅱ: May 2009–February 2022). This distinction is based on the date the additional indication for sorafenib for unresectable HCC was approved in Japan [8]. In other words, sorafenib became available for unresectable HCC in our hospital in period Ⅱ.

### 2. Study endpoint

The endpoint for this analysis was OS which was calculated from hospitalization for treatment at the initial date until death by any cause or the last follow-up. Baseline factors associated with OS were analyzed. The factors analyzed for prognostic significance included age, sex, serum data (albumin, bilirubin, alpha-fetoprotein [AFP] and protein induced by vitamin K absence-II [PIVKA-Ⅱ]), C-P score, and ALBI score, and BCLC stage). Furthermore, we analyzed the OS of patients with each period and prognosis factor.

### 3. Statistical analyses

Chi-squared and Fisher’s exact tests were used to compare categorical data. Student’s t-test or Mann–Whitney U test was used to analyze the distribution of continuous variables. The Kaplan–Meier method was used to prepare cumulative survival rates of patients. The log-rank Mantel–Cox test was used to compare the cumulative survival curves. Cox proportional hazards model was used to analyze the factors associated with prognosis. The Fine-Gray competing risk model was developed to consider competing risks compared with the Kaplan–Meier and Cox model. *P*-values of <0.05 was considered statistically significant. GraphPad Prism software (GraphPad Software, San Diego, CA, USA) and EZR (Saitama Medical Center, Jichi Medical University, Shimotsuke, Japan) were used to analyze statistical significance.

## Results

### 1. Patient characteristics

This study analyzed 566 patients who were diagnosed with HCC through AG from February 2002 to February 2022. Patient characteristics were presented in Table 1. The median age was 72 (range: 30–95) years. Regarding background liver disease etiology, 64 (11.3%), 324 (57.2%), 95 (16.8%), 24 (4.2%), and 59 (10.4%) patients had HBV, HCV, alcoholic liver disease (ALD), non-alcoholic steatohepatitis (NASH), and others. The median ALBI score was −2.477. The number of males was 382 (67.5%), and C-P grades A and B were found in 452 (79.9%) and 109 (19.3%) patients, respectively. BCLC stages 0/A (early) and B (intermediate) were found in 404 (71.4%) and 162 (28.6%) patients, respectively. The distribution of treatment was as follows: resection in 116 (20.4%), radiofrequency ablation (RFA) and/or TACE in 443 (78.2%), systematic therapy in 5 (0.9%), and HAIC in 2 (0.3%). The total number of systematic therapy and HAIC was 22 (3.8%) and 5 (0.9%), respectively, when the post-anticancer treatments after local therapy are included. The median observation duration was 3.49 years.

**Table 1:** Baseline characteristics of patients with nonadvanced hepatocellular carcinoma.

	All (2002/2-2022/2, n = 566)	Period I (2002/2~2009/4, n = 215)	Period II (2009/5~2022/2, n = 351)	P value (I vs II)
Age (years)	72 (30–95)	70 (34–90)	73 (30–95)	0.00138**
Male	382 (67.5%)	146 (67.9%)	236 (67.2%)	0.926
Etiology HBV/HCV/ALD/NASH/other	64/324/95/24/59	25/152/14/4/20	39/172/81/20/39	
ALBI score	-2.477(-3.878–0.489)	-2.331(-3.436–0.489)	-2.531(-3.878- -0.957)	<0.001***
ALBI grade 1/2a/2b/3	234/133/182/17	78/40/87/10	156/93/95/7	<0.001***
Child A/B/C	452/109/5	158/53/4	294/56/1	0.00347**
BCLC 0/A/ B	404/162	153/62	251/100	0.924
BCLC B UT7 IN (%) /BCLC B	59 (36.4%)	22 (35.4%)	37 (37.0%)	0.868
Tumor burden	3.8 (1.6-multi)	4.0 (1.8-multi)	3.7 (1.6-multi)	0.301
AFP (ng/mL)	10.5 (1.1–670000)	21.9(1.1–670000)	7.2(1.6–484000)	<0.001***
PIVKA-Ⅱ (mAU/mL)	29 (0–188000)	35 (7–188000)	28(0–58677)	0.0619
Tx; Resection/RFA/TACE/RFA+TACE/	116/106/161/176/	42/60/44/69/	74/46/117/107	
Systematic therapy/HAIC	5/2	0/0	5/2	
Observation period (years)	3.49 (0.083–20.1)	3.53 (0.092–20.1)	3.37 (0.083–12.8)	0.117

ALBI, albumin-bilirubin; HBV, hepatitis B virus; HCV, hepatitis C virus; ALD, alcoholic liver disease; NASH, non-alcoholic steato hepatitis; BCLC, Barcelona Clinic Liver Cancer; UT7, up to seven; AFP, Alpha fetoprotein; PIVKA. protein induced by vitamin K absence or antagonist;Tx, treatment; RFA, radiofrequency ablation; TACE, transcatheter arterial chemo embolization; HAIC, hepatic arterial infusion chemotherapy"

### 2. Transition of characteristics according to time

Table 1 shows the baseline characteristics of patients with non-advanced HCC subdivided into two groups according to the date of hospitalization (period I: February 2002–April 2009 and period Ⅱ: May 2009–February 2022). Patients in period II (n = 351) were significantly older (median age: 73 vs. 70 years, *P* < 0.01), with lower ALBI scores (median: −2.531 vs. −2.331, *P* < 0.001), lower AFP (median: 7.2 vs. 21.9 ng/mL, *P* < 0.001), and more systematic therapy and HAIC (*P* = 0.048) as compared with those in period I (n = 215). HCV incidence decreased from 152 (70.6%) to 172 (49.0%) and non-HBV and non-HCV (NBNC) increased from 38 (17.7%) to 140 (39.9%) from periods I to Ⅱ.

### 3. The prognosis factors of patients with non-advanced HCC

**Fig 1:**
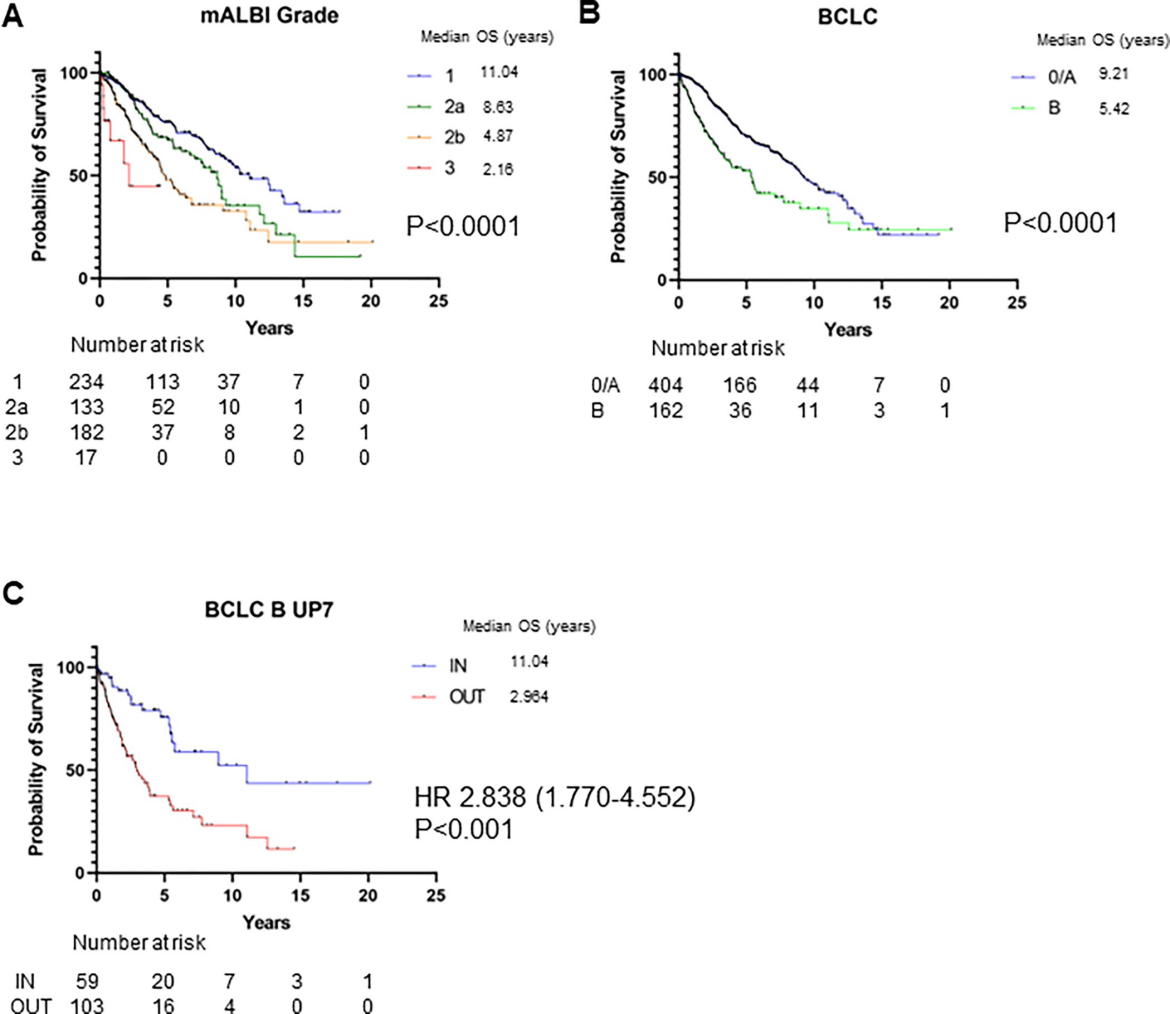
Overall survival of patients with non-advanced HCC. (A) Overall survival assessed by modified ALBI grade. (B) Overall survival assessed by BCLC stage. (C) Overall survival assessed by UT7 criteria in BCLC B-stage.

First, we investigated the OS of patients with non-advanced HCC (whole cohort). Survival curves, according to the liver function score, mALBI grade, and BCLC stage were shown in Fig 1A and 1B, respectively. The median OS in patients was significantly longer for better mALBI grade (grade 1, 11.04 years; grade 2a, 8.63 years; grade 2b, 4.87 years; grade 3, 2.16 years; *P* < 0.001). The median OS of patients with BCLC stage 0/A was significantly longer than those with BCLC stage B (9.21 years vs. 5.42 years, *P* < 0.001). OS was investigated in the prognosis of patients as compared within (IN) and beyond (OUT) up to seven (UT7) in patients with BCLC stage B. The median OS of UT7 IN patients was significantly longer than those with UT7 OUT (11.04 years vs. 2.96 years, *P* < 0.001, Fig 1C). These results revealed liver function, tumor stage, and burden as significant prognostic factors of patients with non-advanced HCC.

Next, we investigated the prognosis factors of patients with non-advanced HCC using univariate and multivariate Cox proportional hazards regression analysis. The univariate analysis revealed age, ALBI score (and C-P grade), AFP, antiviral therapy as significant prognosis factors. The multivariate analysis revealed older age (HR: 1.019; 95% CI: 1.003–1.035; *P* = 0.022), higher ALBI score (HR: 2.25; 95% CI: 1.683–3.007; *P* < 0.001), AFP of >20 ng/mL (HR: 1.687; 95% CI: 1.265–2.25; *P* = 0.003), and antiviral therapy (HR: 0.499; 95% CI: 0.269–0.923; *P* = 0.028) as independent prognosis factors of patients with non-advanced HCC (Table 2).

**Table 2:** Cox proportional hazards regression analysis for factors associated with prognosis of nonadvanced hepatocellular carcinoma.

	Univariate analysis			Multivariate analysis		
	HR	95%CI	P-value	HR	95%CI	P-value
Age	1.019	1.004–1.034	0.01388	1.019	1.0030–1.0350	0.0219*
Male	1.028	0.769–1.374	0.8525			
ALBI score	2.3	1.756–3.014	<0.001	2.25	1.6830–3.007	<0.001***
Child A vs B	2.026	1.469–2.794	<0.001			
AFP>20 ng/mL	2.067	1.568–2.725	<0.001	1.687	1.2650–2.25	0.00373**
Viral hepatitis (HBV, HCV)	1.021	0.7508–1.389	0.8936			
Antiviral therapy	0.4317	0.2352–0.7924	0.0067	0.4993	0.2685–0.9287	0.0282*

ALBI, albumin-bilirubin; AFP, alpha fetoprotein; HBV, hepatitis B virus; HCV, hepatitis C virus

### 4. OS of each BCLC stage in periods I or Ⅱ in crude analysis

**Fig 2:**
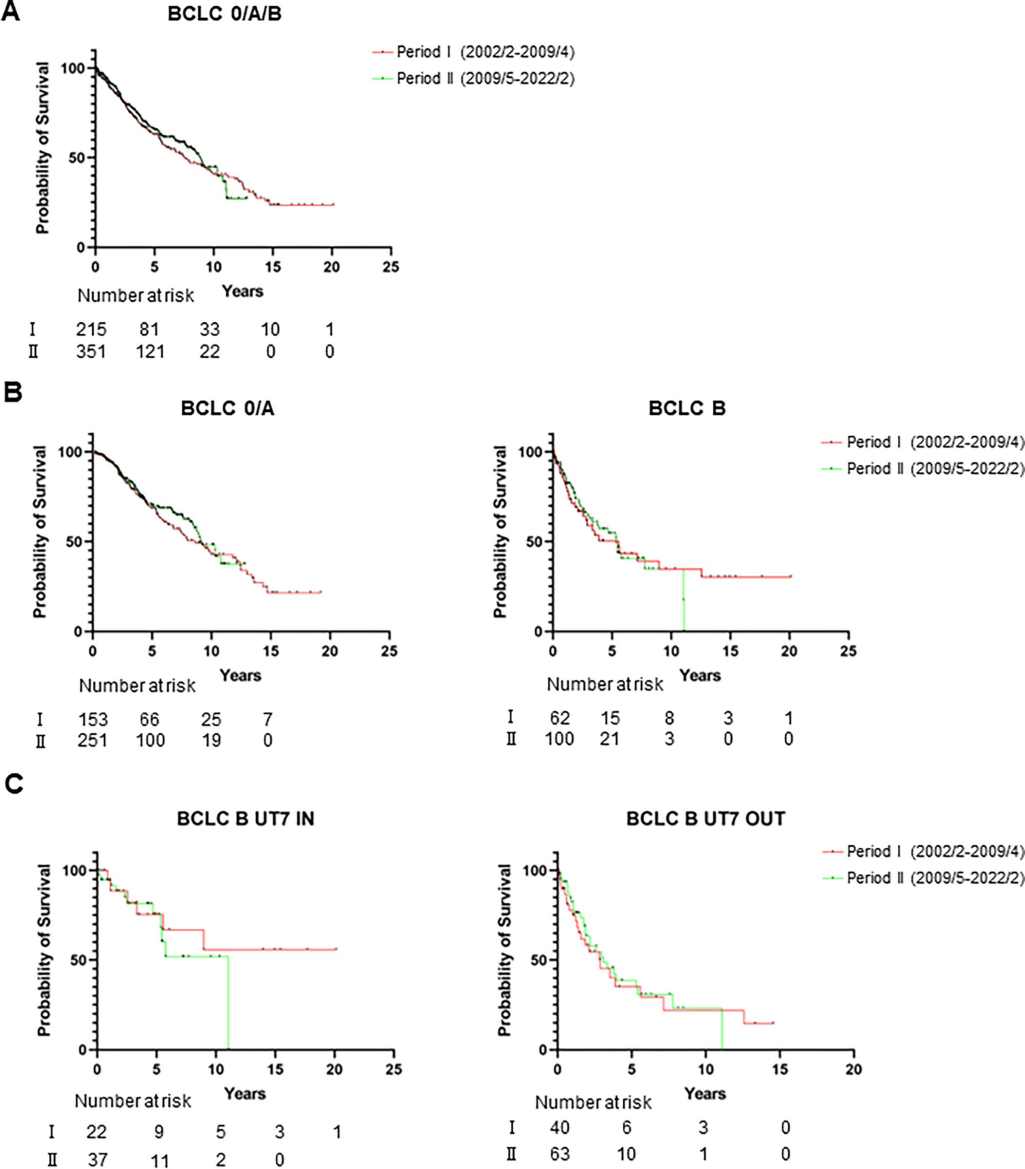
Overall survival of patients compared with periods I and Ⅱ. (A) Overall survival in patients with BCLC 0/A/B assessed by period. (B) Overall survival in patients subdivided with BCLC 0/A and B assessed by period. (C) Overall survival in patients subdivided by UT7 criteria in BCLC B-stage assessed by period. *period was shown as (year/month).

Median OS of patients with non-advanced HCC in periods I (n = 215) and Ⅱ (n = 351) was 7.65 years and 8.91 years, respectively (log-rank test, *P* = 0.568, Fig 2A). No significant difference was found in the median OS of patients with subdivided stage 0/A and B between periods I and Ⅱ (0/A: 8.63 years vs 9.21 years, *P* = 0.568; B: 5.55 years vs 5.35 years, *P* = 0.931, respectively, Fig 2B). No significant difference was found in the median OS of patients with BCLC B subdivided UT7 IN and OUT between periods I and Ⅱ focused on the BCLC B-stage with UT7 criteria (IN: undetermined vs. 11.04 years, *P* = 0.45; OUT: 2.88 years vs. 3.10 years, *P* = 0.736, respectively, Fig 2C).

### 5. Analysis in the prognosis of each BCLC 0/A and B stages with the comparison between period I and Ⅱ

#### BCLC stage 0/A

Regarding the baseline characteristics, patients in BCLC stage 0/A in period II (n = 251) were significantly older (median age: 73 vs. 71 years, *P* = 0.048), with lower ALBI scores (median: −2.558 vs. −2.388, *P* = 0.0028), lower AFP (median: 6.5 vs. 20.7 ng/mL, *P* < 0.001), and more antiviral therapy than those in period I (n = 153) (Table 3A). The multivariate Cox proportional hazards regression analysis revealed older age (HR: 1.027; 95% CI: 1.005–1.049; *P* = 0.015) and higher ALBI score (HR: 2.18; 95% CI: 1.536–3.093; *P* < 0.001) as independent prognosis factors of patients with BCLC stage 0/A (Table 4A). These results revealed that lower ALBIs have a positive effect on prognosis in patients in period II. Conversely, older age may negatively affect prognosis, and therefore no significant improvement was found in prognosis by period (Fig 2B).

**Table 3:** Baseline characteristics of patients with hepatocellular carcinoma in BCLC stage 0/A or B.

**A.**			
BCLC 0/A stage	Period I (2002/2~2009/4, n = 153)	Period II (2009/5~2022/2, n = 251)	P value (I vs II)
Age (years)	71(42–88)	73(38–89)	0.048*
Male	97 (63.4%)	164 (65.3%)	0.748
ALBI score	-2.388(-3.436- -0.902)	-2.558(-3.636- -0.957)	0.00279**
ALBI grade 1/2a/2b/3	60/30/59/4	118/65/64/4	0.0309*
Etiology HBV/HCV/ALD/NASH/other	18/116/6/2/11	23/134/57/14/23	0.146
Child A/B/C	115/36/2	210/40/1	0.0632
Tumor burden	3.3 (1.8–5.8)	3.0 (1.6–5.9)	0.078
AFP (ng/mL)	20.7(1.6–5660)	6.5(1.6–2520)	<0.001***
PIVKA-Ⅱ (mAU/mL)	26(7–31900)	23(0–28785)	0.109
Tx; Resection/RFA/TACE/RFA+TACE/Systematic therapy/HAIC	23/57/20/53/0/0	53/46/69/83/0/0	
Antiviral therapy	0	36 (HCV 29 /HBV 7)	<0.001***
**B.**			
BCLC B stage	Period I (2002/2~2009/4, n = 62)	Period II (2009/5~2022/2, n = 100)	P value (I vs II)
Age (years)	67(34–90)	72(30–95)	0.0044**
Male	49(79.0%)	72(72.0%)	0.357
ALBI score	-2.251(-3.113- -0.489)	-2.475(-3.878- -1.035)	0.0176*
ALBI grade 1/2a/2b/3	18/10/28/6	38/28/31/3	0.0398*
Etiology HBV/HCV/ALD/NASH/other	7/36/8/2/9	16/38/24/6/16	0.146
Child A/B/C	43/17/2	84/16/0	0.0276*
Tumor burden	7.7 (4.9- multi)	7.9 (4.5-multi)	0.427
AFP (ng/mL)	27.3(1.1–670000)	12.4(1.9–484000)	0.0133*
PIVKA-Ⅱ (mAU/mL)	333(9–188000)	104(10–58577)	0.151
Tx; Resection/RFA/TACE/RFA+TACE/Systematic therapy/HAIC	19/3/24/16/0/0	21/0/48/24/5/2	
Antiviral therapy	3 (HCV1/ HBV2)	19 (HCV10/ HBV9)	0.00988**

ALBI, albumin-bilirubin; HBV, hepatitis B virus; HCV, hepatitis C virus; ALD, alcoholic liver disease;NASH, non-alcoholic steato hepatitis; BCLC, Barcelona Clinic Liver Cancer; UT7, up to seven; AFP, Alpha fetoprotein; PIVKA. protein induced by vitamin K absence or antagonist; Tx, treatment

**Table 4:** Cox proportional hazards regression analysis for factors associated with prognosis of hepatocellular carcinoma in BCLC stage 0/A or B.

**A.**						
BCLC 0/A stage	Univariate analysis			Multivariate analysis		
	HR	95%CI	P-value	HR	95%CI	P-value
Age	1.031	1.01–1.052	0.00298	1.027	1.005–1.049	0.01463*
Male	0.9068	0.6445–1.276	0.5743			
ALBI score	2.315	1.674–3.203	<0.001	2.18	1.536–3.093	<0.001***
Child A vs B	2.08	1.392–3.109	<0.001			
AFP>20 ng/mL	1.714	1.219–2.41	0.00196	1.403	0.9832–2.002	0.0695
Viral hepatitis (HBV, HCV)	1.2	.7865–1.83	0.398			
Antiviral therapy	0.306	0.1133–0.8296	0.01992	0.4072	0.1495–1.109	0.078
Tx selection (TACE)	1.303	0.9353–1.816	0.1176			
**B.**						
BCLC B stage	Univariate analysis			Multivariate analysis		
	HR	95%CI	P-value	HR	95%CI	P-value
Age	1.006	0.985–1.028	0.5609			
Male	1.163	0.6551–2.065	0.606			
ALBI score	2.051	1.254–3.353	0.0041	2.417	1.468–3.978	<0.001***
Child A vs B	2.013	1.173–3.455	0.011			
AFP>20 ng/mL	2.617	1.589–4.31	<0.001	2.198	1.301–3.714	<0.001***
Viral hepatitis (HBV, HCV)	1.074	0.6649–1.1736	0.7694			
Antiviral therapy	0.4943	0.2242–1.09	0.08			
Tx selection (TACE)	1.078	0.6491–1.79	0.772			

ALBI, albumin-bilirubin; AFP, alpha fetoprotein; HBV, hepatitis B virus; HCV, hepatitis C virus; Tx, treatment

**Fig 3:**
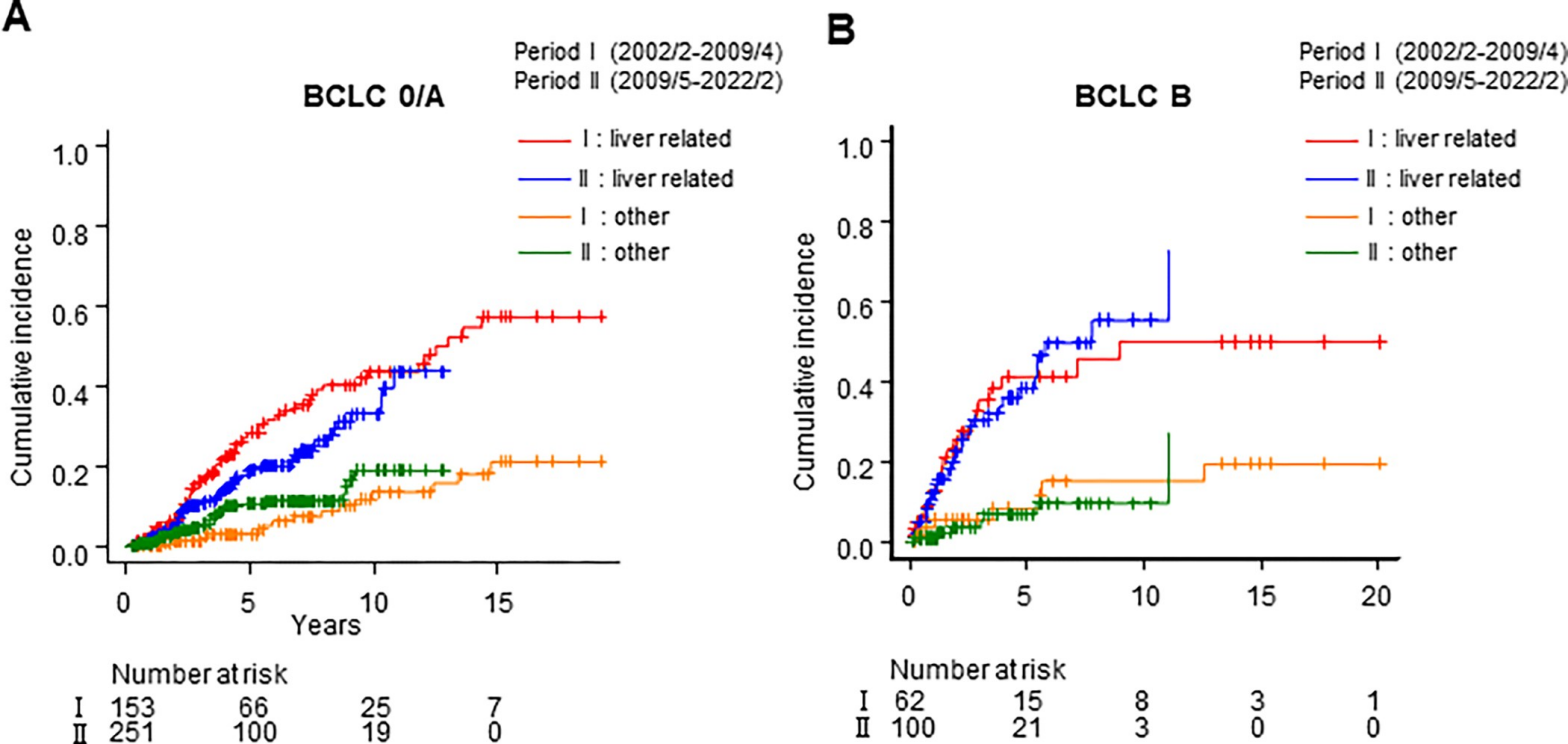
Cumulative incidence of death in patients with HCC through competing risk model analysis (liver-related and others). (A) patients with BCLC stage 0/A assessed by period. (B) patients with BCLC stage B assessed by period. *period was shown as (year/month).

Moreover, we investigated liver-related and other causes of death. A total of 143 patients died during observation periods. The number of liver-related death and others was 103 and 40, respectively. Age adjusted Fine-Gray competing risk model analysis revealed decreased liver-related deaths in period II as compared to period I (HR: 0.656, 95% CI: 0.442–0.972, *P* = 0.036, Fig 3A).

#### BCLC stage B

The baseline characteristics of patients in BCLC stage B in period II (n = 100) were significantly older (median age: 72 and 67 years, *P* = 0.0044), with lower ALBI scores (median: –2.475 and –2.251, *P* = 0.018), lower AFP (median: 12.4 and 27.3 ng/mL, *P* = 0.013), and more antiviral therapy than those in period I (n = 62) (Table 3B). The multivariate Cox proportional hazards regression analysis revealed higher ALBI scores (HR: 2.18; 95% CI: 1.536–3.093; *P* < 0.001) and AFP of >20 ng/mL (HR: 2.198; 95% CI: 1.301–3.714; *P* < 0.001) as independent prognosis factors of patients with BCLC stage B (Table 4B). Age adjusted Fine-Gray competing risk model analysis revealed no significant difference in liver-related deaths between periods I and Ⅱ (*P* = 0.62, Fig 3B).

### 6. Antiviral therapy for viral hepatitis as background liver disease

Additionally, we investigated the effects of antiviral therapy on background liver management based on the BCLC stage. Among 388 patients with viral hepatitis, a total of 58 patients have received antiviral therapy. The median OS of patients with viral hepatitis background who were treated with antiviral therapy was significantly longer (HR: 0.4162, 95% CI: 0.269–0.642, *P* = 0.036, S1A Fig), especially in BCLC 0/A (HR: 0.286, 95% CI: 0.160–0.512, *P* = 0.0084, Sup S1B Fig left panel). Whereas there was no significant difference in BCLC B stage (*P* = 0.06, S1B Fig right panel). Though patients in period II had more antiviral therapy than period I, the number of patients with administrated antiviral therapy after treatment for HCC decreased as the disease stage progressed especially in HCV (S1 Table).

## Discussion

### Main findings

This study revelated the actual transition and changes in the prognosis and background liver disease of non-advanced HCC in the past 2 decades. Patients with HCC were older, with lower ALBI, better liver function, lower AFP, and more antiviral therapy for viral hepatitis as time goes from period I to Ⅱ. Viral hepatitis (HBV and HCV) decreased and non-viral hepatitis increased as the etiology of HCC (Table 1).

The prognostic factors included age and ALBI (Table 4A), and were more dependent on the status of the background liver disease rather than the tumor status in BCLC stage 0/A. Some competing factors, such as older age, antiviral therapy, and better liver function, were observed through the transition from periods I to Ⅱ. Fine-Gray competing risk model analysis showed a decreasing in liver-related deaths of BCLC stage 0/A in period Ⅱ (Fig 3A). Non-liver-related death is increasing due to older age, which may explain why the overall prognosis does not appear to be improving in crude OS analysis.

Prognostic factors included ALBI and AFP in BCLC stage B (Table 4B), which had a greater influence from tumor burden, in addition to the background liver function. These results revealed that BCLC B HCC prognosis was determined not by age factor but by HCC tumor burden and liver function, which differs from BCLC 0/A stage. However, OS has not significantly improved, despite improvement in prognostic factors, such as AFP and ALBI, in BCLC B-stage period II (Fig 3B).

### Context with published literature

A nationwide survey report on HCC in Japan from 2011 to 2015 reported that patients with HCC were older (median ages: 70–73 years) and more obese. and the proportions of patients with diabetes mellitus, hypertension, dyslipidemia, and fatty liver (24.0%–28.8%) had all significantly increased as compared previous report [22]. Non-liver-related death is frequent after HCV- sustained viral response (SVR), and we should focus on the management of extrahepatic manifestations of HCV [23]. These data and our analysis of liver and non-liver-related deaths in the BCLC 0/A stage suggest that the management of non-liver-associated deaths is necessary to improve OS, especially for BCLC 0/A stage. Furthermore, we referenced the survival rates of the general population [24]. The mean age of males and females in the BCLC0/A was 69 and 72 years, respectively. The 10-year survival rates for the general population were 47.0% for males and 49.6% for females, respectively. These rates were comparable to 48.1% and 45.9% observed in patients with BCLC stage 0/A in period II. The general Japanese population database used as a reference group was provided by the National Cancer Center Japan (https://ganjoho.jp/reg_stat/statistics/qa_words/cohort01.html). The proportion of patients with NBNC increased from 10.0% in 1991 to 32.5% in 2015[22]. The distribution of initial treatments (resection: 21.7%, ablation: 18.9%, ablation+TACE: 9.0%, TACE: 31.4%, TAI or HAIC: 7.0%, and systemic therapy: 2.8%) was similar to our study, and HAIC and systemic therapy were less due to our cohort limited in the non-advanced stage. Our study was considered to be a cohort that reflects the overall trends in Japan, because of such background liver disease trends and treatment distribution.

Our study revealed no significant difference in OS of patients with the non-advanced stage HCC. This result was supported by the report on patients who received any MTAs as first-line systemic therapy for advanced HCC from June 2009 to March 2019 (period 1: 2009–2012, period 2: 2013–2016; period 3: 2017–2019) [18]. The OS of patients with non-advanced stage HCC were not significantly different between the 3 periods although the proportion of patients who received multiple MTAs remarkably increased over time. Conversely, the median OS of patients with advanced stage HCC was prolonged to 10.4, 11.3, and 15.2 months in periods 1, 2, and 3, respectively [18]. Additionally, proving the optimal treatment strategy, even in the BCLC B-stage with multiple MTAs, remains difficult.

The prognosis of patients with BCLC B-stage was better with systemic therapy in refractory TACE cases rather than with TACE disorderly repetition [15, 25]. In particular, the prognosis of patients with UT7 OUT is prolonged with systemic therapy [26]. The number of patients with BCLC B UT7 OUT in period Ⅱ of our cohort (14.2%) who were administrated systemic therapy might not be enough. Additionally, no significant difference was found in baseline ALBI score, when the tumor burden rises higher to BCLC UT7 OUT. This cohort mainly consisted of patients with HCV, including BCLC B-stage UT7 OUT. Anti-HBV therapy provides significantly better survival in patients not only with curative resection or RFA [27–30] but also irrespective of baseline viremia status or tumor stage [31–33], regardless of initiation before or after HCC diagnosis. Conversely, antiviral therapy for HCV in patients with more advanced BCLC stages remains a controversial topic, different from HBV-HCC [34]. HCV eradication needs cancer-free status in present Japanese regulation.

The age factor should be particularly considered in our study. Age was not a prognostic factor in the BCLC B-stage, but patients in each stage were significantly older. Generally, only approximately 20%–30% of HCC are diagnosed at an early stage, and almost half of all patients with HCC receive systemic therapies because of the high recurrence rate even after successful treatment, such as tumor resection and ablation [35]. From this point of view, elderly patients who undergo systemic therapy for HCC may increase. The decision tree analysis revealed that systemic sequential therapy prolonged the OS of unresectable HCC, especially for patients with good hepatic function and non-advanced age [36]. Additionally, advanced age is a significant factor that causes discontinuation of systemic therapy due to adverse events [37]. These reports revealed that the benefits of anti-HCC systemic therapy for elderly patients should be well-considered. The clinical usefulness of geriatric assessment in elderly patients who received TKIs was reported, which consisted of age, food intake, weight loss, mobility, neuropsychological problems, body mass index, prescription drugs, and a self-perception of health using a Mini-Nutritional Assessment questionnaire [38]. Optimization of treatment may be necessary for elderly patients.

### Strengths and limitations

This is the first study that revealed prognosis and associated factors in patients with each BCLC stage of non-advanced HCC from analysis of OS and background liver disease in two decades of transition. Angiography which included CT during hepatic arteriography and arterial portography was performed mainly prior to 2008 (EOB-MRI was not available) to obtain a definitive HCC diagnosis. Therefore, the diagnosis of HCC became more accurate and it may contribute to sophisticated analysis. Clinical characteristics and courses in each case were closely scrutinized because of a well-managed single-center cohort.

Liver-related death decrease from periods I to Ⅱ in BCLC stage 0/A. The effort of intervention for background liver disease shows effectiveness. Furthermore, the improvement in OS due to decreased liver-related deaths was offset by an increase in non-liver-related deaths due to the aging of patients with HCC. This suggests success in follow-up for recurrence and appropriate therapeutic management for background liver disease and difficulty in the management of non-liver-related death of elderly patients. Moreover, the prognosis of patients with BCLC B-stage did not significantly improve from period I to Ⅱ. Further investigation for treatment strategy of locoregional therapy, systemic, and ICI is warranted to improve the prognosis of patients with BCLC stage B, especially UT7 OUT. To our best knowledge, no report has analyzed background liver disease, anti-cancer treatment, and historical background of non-advanced HCC in detail, as this study.

Our study also had some limitations. First, ruling out bias that cannot be identified to some extent is impossible because this is a retrospective study. The characteristic of this cohort partially limited the solid analysis in systemic therapy and whether antiviral therapy pre or post HCC therapy due to small number of these patients. Even in BCLC B OUT of period II, TACE remained a main treatment (45 cases: 71.4%). Systemic therapy was administrated after 2017 in the later period II. Based on these data, the concept of BCLC UT7 seems to have started gaining traction not with the introduction of SOR but rather in the latter part of period II. For antiviral therapy, the number of cases with administrated after treatment for HCC decreased as the progression of the disease stage, as complete cure is required for HCV (S1 Table). Although it is difficult to fully evaluate the timing and effectiveness of induction because of the insufficient number of these patients and the results were qualified by the restriction of curative nature in anti-HCV. Second, not all patients underwent biopsies of background liver disease and/or tumor. This was a limitation of our study since it prevented definitive confirmation of all background liver disease and detail for HCC. Third, our results were exclusively drawn from a Japanese population, possibly limiting their generalizability to other ethnicities. Further, international, research, and validation studies are required.

### Future implications

This study highlights the success of background liver disease management and antiviral therapy and the need for further investigation of BCLC B-stage treatment. Undoubtingly, hepatic reserve function is important at all stages. This study suggests two important points in helping patients be cancer-free or maintaining liver function and continuing anti-cancer treatment in patients with BCLC B stages. An appropriate decision is necessary on a case-by-case basis, whether to use TACE, MTAs, ICI, combination, or conversion therapy [39, 40].

The indication for DAA for HCV-HCC is only for cancer-free patients, but this may change with an accumulation of evidence that DAA improves BCLC B prognosis. The treatment for patients with BCLC B UT7 OUT with systemic therapy to maintain liver function is becoming more widespread in the past few years [16, 17, 19]. The number of patients who were administrated systemic therapy might be inadequate. The positive results are emerging, and it may take several more years to become clear.

Conversely, patients with HCC are going to become older. The decision will be more difficult in the case of advanced HCC treatment in the future although age itself is a risk of cancer. Elder patients should manage not only the HCC which is detected at an early stage but also extrahepatic complications. The identification of a strategy for individuals with a high risk of developing HCC and extrahepatic manifestations is needed to further improve patient survival, even in the case who received curative anti-HCC or background liver disease therapy, especially for the early stage.

In conclusion, the background characteristics of HCC have changed over time. Patients with HCC in period II were older, with more antiviral therapy, lower AFP, and better hepatic reserve function. Especially in patients with BCLC stage 0/A, liver-related prognosis was improved. Appropriate background liver management and treatment selection may lead to a better prognosis for patients with HCC.

## Supporting information

S1 Checklist STROBE statement. (DOCX)

S1 Fig Overall survival of patients with viral hepatitis B and C compared with or without antiviral therapy. (A) Overall survival in patients with viral hepatis B and C assessed by antiviral therapy. (B) Overall survival in patients subdivided with BCLC 0/A and B assessed by antiviral therapy.
(TIF)

S1 Table Breakdown of antiviral therapy with period and BCLC stage. (DOCX)
