# Supplementary figures and images for: Analysis of prognosis and background liver disease in non-advanced hepatocellular carcinoma in two decades
Source: PLoS One. 2024 Mar 7;19(3):e0297882. doi: 10.1371/journal.pone.0297882 (PMC10919582; doi:10.1371/journal.pone.0297882)

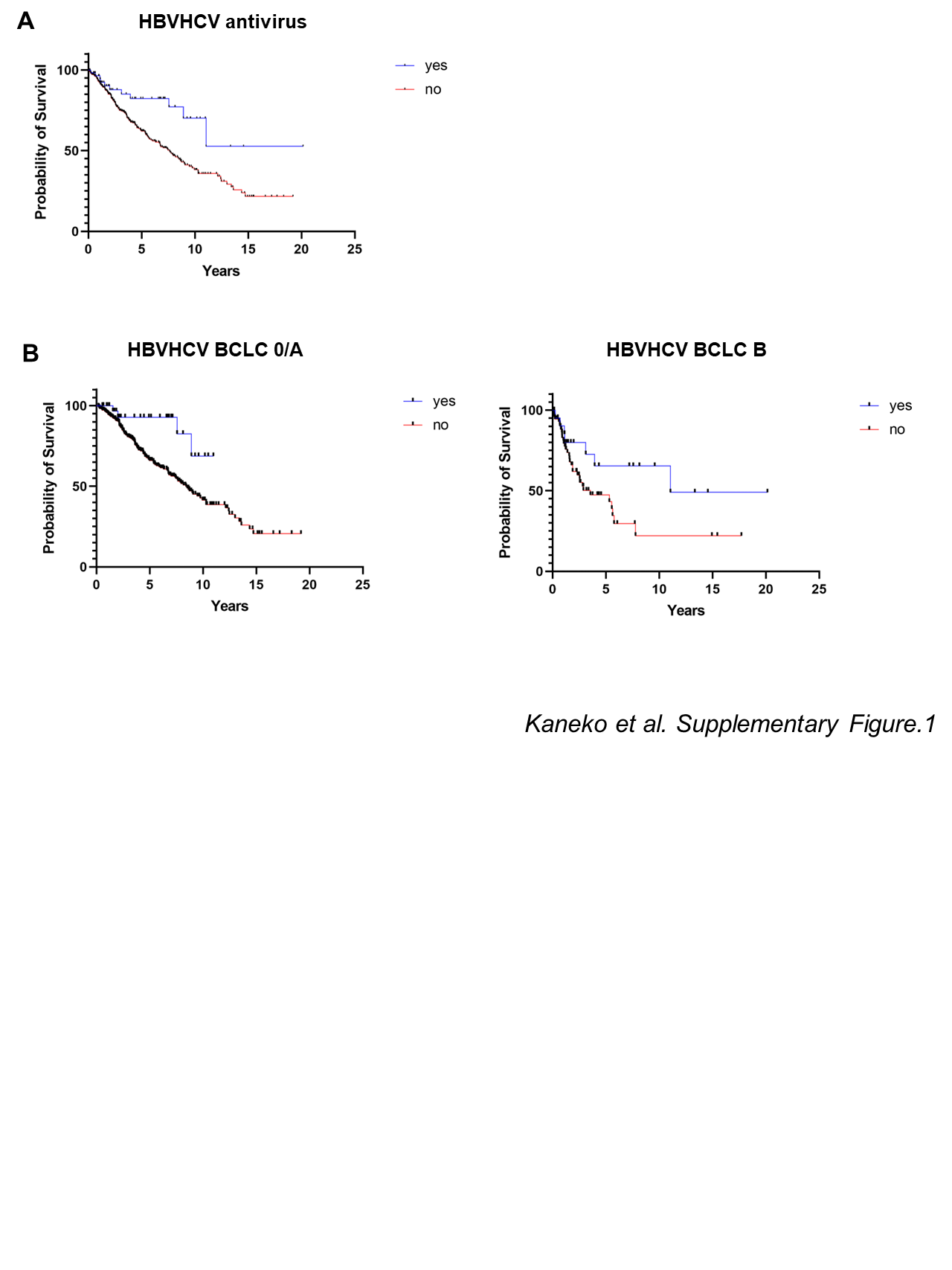

Supplement: S1 Fig — (A) Overall survival in patients with viral hepatis B and C assessed by antiviral therapy. (B) Overall survival in patients subdivided with BCLC 0/A and B assessed by antiviral therapy. (TIF) [file pone.0297882.s002.tif]
