# Supplementary material for: Analysis of prognosis and background liver disease in non-advanced hepatocellular carcinoma in two decades
Source: PLoS One. 2024 Mar 7;19(3):e0297882. doi: 10.1371/journal.pone.0297882 (PMC10919582; doi:10.1371/journal.pone.0297882)
Supplement: S1 Table — (DOCX) [file pone.0297882.s003.docx]

**Supporting information**

Supplementary Table 1. Breakdown of antiviral therapy with period and BCLC stage

|  | | Period I (2002/2～2009/4) | | Period II (2009/5～2022/2) | |
| --- | --- | --- | --- | --- | --- |
| BCLC stage | Timing of antiviral Tx | Pre HCC Tx | Post HCC Tx | Pre HCC Tx | Post HCC Tx |
| 0/A | HBV | 0 | 0 | 7 | 0 |
|  | HCV | 0 | 0 | 20 | 9 |
| B UT7 IN | HBV | 1 | 0 | 0 | 0 |
|  | HCV | 0 | 0 | 2 | 4 |
| B UT7 OUT | HBV | 0 | 1 | 7 | 2 |
|  | HCV | 0 | 1 | 4 | 0 |

BCLC, Barcelona Clinic Liver Cancer; UT7, up to seven; Tx, treatment, HCC, hepatocellular carcinoma; HBV, hepatitis B virus; HCV, hepatitis C virus
